# Biomechanical Changes in Gait Patterns of Patients with Grade II Medial Gonarthritis

**DOI:** 10.3390/diagnostics11071242

**Published:** 2021-07-12

**Authors:** Dmitry Skvortsov, Sergey Kaurkin, Alexey Prizov, Aljona Altukhova, Alexander Troitskiy, Fedor Lazko

**Affiliations:** 1 Scientific Department, Federal Research and Clinical Centre of Russia’s Federal Medical-Biological Agency (FNKC FMBA), Orekhoviy Bulvar, 28, 115682 Moscow, Russia; kaurkins@bk.ru (S.K.); altukhova.aa@bk.ru (A.A.); info@fnkc-fmba.ru (A.T.); 2 Medical Rehabilitation Chair, Pirogov Russian National Research Medical University (RNRMU), Ostrovitianova St. h.1, 117997 Moscow, Russia; 3 Orthopedic Department, Buyanov V.M. Moscow City Clinical Hospital, Bakinskaya St, h. 26, Bild. 10, 115516 Moscow, Russia; aprizov@yandex.ru (A.P.); fedor_lazko@mail.ru (F.L.); 4 Ortopedic Chair, Peoples Friendship University of Russia (RUDN University), Miklukho-Maklaya Str. 6, 117198 Moscow, Russia

**Keywords:** knee joint, medial arthritis, biomechanics, gait analysis

## Abstract

Deforming osteoarthritis (OA) of the knee is a progressive disease associated with pain and compromised function of the joint. Typical biomechanical modifications in the gait of subjects with medial knee OA are characterized by decreased gait speed and overload on the affected limb. The borderline stage for conservative versus surgical management is Grade II OA. The aim of this research was to study preoperatively the specific features of gait, knee, and hip function in patients with Grade II medial OA. We examined 26 patients with Grade II unilateral gonarthritis with varus deformity and 20 healthy adults. Biomechanical parameters of gait were recorded using an inertial sensor system. The gait cycle (GC) slightly increased both for the affected and for the intact limb. The hip joint movements showed significant symmetrical reduction in the first flexion amplitude, as well as a symmetrical delay in full hip extension at the end of the stance phase. In the knee, the first flexion amplitude was significantly reduced on the affected side compared to healthy control. The extension amplitude in the single support phase was significantly increased in both the affected and the intact lower limbs. The swing amplitude was significantly reduced on the affected side. On the affected side, the changes were more pronounced, both in incidence and in severity. The affected knee showed a syndrome of three reduced amplitudes. In patients, walking is characterized by several groups of symptoms: those of unloading of the affected limb, those of limiting the load on the affected joint and the musculoskeletal system as a whole, and those of gait harmonization. The symptoms of unloading the affected side and those of harmonization are the common symptoms of adaptation, typical for several pathological conditions with a relatively preserved function. The intensity of the observed symptoms can help assess changes in the subject’s functional condition over time and during the treatment.

## 1. Introduction

The knee joint is most susceptible to the development of osteoarthritis (OA) [1,2]. Deforming OA of the knee is a progressive disease associated with pain and limited function of the joint [3]. About four-fifths of all OA cases are knee OA [4]. The prevalence of symptomatic gonarthritis is 1020 per 100,000 persons [5]. Deforming arthritis can develop as a consequence of knee trauma or can be caused by some underlying disease, hormonal disorders, or bad habits. Abnormal changes in the anatomical structure, such as loss of articular cartilage and formation of osteophytes, compromise the joint function and worsen the quality of life [6]. On the whole, the available data show a much higher prevalence of radiological OA compared to symptomatic OA [3]. Abnormal loading of the joint is an important factor in the development of OA. The location of the affected area largely depends on the predominant position of the resulting force vector in the medial or lateral part of the knee [7], with the medial location being more common [8]. During daily activities (walking, climbing stairs, running), the femorotibial load can significantly exceed the knee load from body weight when standing in a static position [9]. About 75% of the joint load passes through the medial tibial plateau when leaning on one leg while standing [10]. Due to the specific features of the joint, primary (genuine) arthroses, i.e., those with no identified cause, often begin in the medial compartment as the one that experiences greater loads. The subsequent changes in the knee structures lead to genu varum which adds load to the already degenerated compartment [10]. Here, there is a clear feedback mechanism. A greater lesion of the medial compartment results in a greater load and faster destruction of the knee joint and its medial structures. The changes in the knee anatomy are followed by rearrangement of its functional and motor activity, thus leading to changes in walking kinematics [11].

As an objective research method, the study of knee biomechanics is of considerable clinical interest [12]. The function of movement and load weightbearing are the basis of the knee joint activity. Changes in gait are a simple and primary strategy implemented by the body to compensate for knee pain due to medial gonarthritis. Increased toe dorsiflexion and lateral tilt of the trunk (or mediolateral position of the trunk), as well as the use of walking aids, help relieve the pain syndrome [13].

Typical biomechanical gait modifications associated with medial knee OA are characterized by a lower gait speed, which reduces the load on the affected limb, as well as smaller flexion–extension amplitudes in the affected knee [14]. The load on the medial knee compartment is usually estimated indirectly, using biomechanical methods, since its direct measurement is invasive. For medial gonarthritis, the most important parameter is external knee adduction moment [15]. While walking, additional dynamic adduction moment emerges in the knee during the single support phase. [16]. The cadence and stride length decrease, whereas the duration of the double support phase (DS) increases symmetrically on both sides [17].

Thus, the walking function and, especially, the knee movement kinematics in medial OA are not well enough studied, in contrast to the basic temporal and spatial characteristics of gait.

One of the methods for treating early medial OA is valgus-producing high tibial osteotomies, which, by correcting the limb axis, move the loads laterally from the medial compartment [18]. In such cases, decisions on the choice of the method and the scope of surgery, as well as the outcome assessment, are made on the basis of anatomical data alone, whereas the function of the affected joint remains beyond the decision-making criteria.

## 2. Aim of Research

The aim of our research was to preoperatively study the gait patterns and knee and hip function in subjects with Grade II medial OA in order to identify the specific features of the affected joint functioning and the developing compensatory changes.

## 3. Materials and Methods

This research presents the investigation of 26 patients with Grade II unilateral medial gonarthritis according to the Kellgren and Lawrence scale, with varus deformity. The studies were carried out at the Federal Scientific and Clinical Center of the FMBA and the Moscow City Clinical Hospital named after V.I. V.M. Buyanov. The patient data are presented in Table 1.

Standing weightbearing teleroentgenograms were obtained from all the patients. The varus angle of the affected limb [19] was measured according to X-ray images and ranged from 3° to 10.3° (mean = 6.52°).

The mechanical lateral distal femoral angle (mLDFA) and the mechanical medial proximal tibial angle (mMPTA) were also measured [19]; mLDFA ranged from 85.9° to 90°, mean = 88.16°, whereas mMPTA ranged from 82.9° to 87.4°, mean = 84.73°.

Knee function was assessed using the Knee Injury and Osteoarthritis Outcome Score (KOOS) [20]. The mean score was 43.76, range 14–81.

MRI scans of the knee were obtained from all patients. Meniscal lesion was assessed according to the Stoller classification [21]. Medial meniscal tear was detected in 22 (84.6%) patients, and four (15.4%) patients had a history of arthroscopic resection of the medial meniscus due to Grade IIIb tear. Grade II medial meniscal tear was found in nine (34.6%) patients, along with Grade III in five (19.2%) patients and Grade IIIb in eight (30.8%) patients. Lateral meniscal tear was detected in 12 (46.2%) patients: Grade II in five patients (19.4%) Grade I in seven patients (26.9%*).*

The pain syndrome was assessed using a Visual Analogue Scale (VAS) [22] and ranged from 4–9 (mean = 6.73).

The study population did not include patients with any lesions of the knee ligaments, pronounced knee instability, medial gonarthritis Grades 0–1 and 3–4 according to Kellegren-Lawrence, patients after arthroscopic suture of the meniscus or syndesmoplasty of the knee joint, or patients with patellar cartilage lesions with chondromalacia of more than ICRS Grade 2 [23].

The control group included 20 healthy adults with normal weight condition, 14 males and six females. Their mean age was 29.7 years. The basic parameters of walking remain stable from adolescence to 70 years of age [24,25]. Therefore, it is permissible for a group of healthy people to be younger. Moreover, parameters such as stride length and walking speed change with age [25], which were not recorded in this study.

The study was approved by the Local Ethics Committee of FNKC FMBA, number 06–07.04.17. All subjects gave written informed consent before the tests.

**Figure 1:**
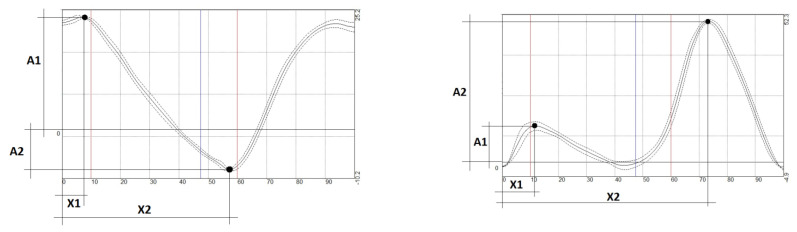
Measured amplitudes (A) and phases (X) in hip (**left graph**) and knee (**right graph**) goniograms are shown in the average graphs.

Biomechanical parameters of gait were recorded using an inertial sensor system (‘Trust-M’ by Neurocor Ltd., Moscow, Russia). Five sensors were applied at the sacrum, the middle third of the thigh, and the outer ankles of both legs. Gait analysis was performed while walking on a flat surface (floor) at an arbitrary pace. The test lasted 30 to 60 s to capture the necessary number (20–30) of gait cycles to calculate mean parameters with a relatively small standard deviation. Defective strides, stops, and turns were excluded from the analysis [24]. Temporal, dynamic, and kinematic parameters were recorded. The temporal parameters included GC (gait cycle duration, in s) and SDS (period from the start of the GC of one leg until the other leg comes into contact with the support (after swing), % of GC). The dynamic parameter was the amplitude of impact load at the beginning of stance phase (at the contact with the support) and was measured in *g* (acceleration due to gravity). The kinematic parameters were the amplitudes and phases of hip and knee movements (Figure 1) and were measured in the primary movement direction, i.e., in the sagittal plane (flexion-extension).

For the hip joint, we recorded maximum flexion amplitude at the start of SP (A1, °) and its phase (X1, % of GC), as well as maximum extension amplitude at the end of SP (A2, °) and its phase (X2, % of GC).

For the knee joint, we measured the amplitudes and phases of the first flexion (A1 and X1, respectively), extension (A2 and X2, respectively), and second flexion (in the swing period) (A3 and X3, respectively).

In the abduction—adduction and rotation planes, we recorded the total range of motion.

The obtained data were processed using standard ANOVA methods of the Statistica 12 package. The numerical data are presented as medians and quartiles (25th percentile, 75th percentile). As the sample size was small, no normality tests were performed, and the significance of the differences was assessed using the Wilcoxon–Mann–Whitney test with *p* < 0.05. Comparative assessments of similar study parameters were done for affected and healthy sides versus healthy control.

## 4. Results

The results of the biomechanical study are given in Table 2, Table 3 and Table 4. The changes in temporal parameters are presented below (Table 2).

The gait cycle significantly increased both for the affected (*p* = 0.047) and for the intact (*p* = 0.046) limb. The SDS data showed minor, albeit significant, differences for the affected limb only (effect of unloading the side). Impact loads remained normal.

The results obtained for the hip joint (Table 3) demonstrate a highly significant symmetrical reduction in the first flexion amplitude (A1), as well as a symmetrical delay in full hip extension at the end of the stance phase (X2). Adduction–abduction (Add.) and rotation (Rot.) movements did not change.

In the knee (Table 4), the first flexion amplitude (A1) at the start of the stance phase was significantly reduced on the affected side compared to healthy control. The extension amplitude in the single support phase was significantly increased in both the affected and the intact lower limbs (A2). The swing amplitude (A3) was significantly reduced on the affected side. The phase of amplitude was also significantly increased on both sides.

The adduction–abduction movements (Add.) on both affected and unaffected sides were significantly reduced compared with control. The rotational movements (Rot.) on the affected and unaffected sides showed no significant changes.

## 5. Discussion

The preoperative study of gait biomechanics in patients with Grade II medial knee OA found a set of persistent and stereotypical changes in the knee and hip function on both the affected and the healthy sides. On the affected side, the changes were more pronounced, both in incidence and in severity.

GC was increased symmetrically for both lower limbs. This is an indirect symptom of walking speed reduction. In the study population, however, the changes were small and could only be detected instrumentally.

The SDS reduction on the affected side was also small. The symptom of unloading is due to an earlier start of the contralateral limb GC. A similar result was obtained in our previous study of acute-phase anterior cruciate ligament tear [24].

The reduced hip flexion amplitude on both sides indicates a likely decrease in the stride length, i.e., its dependent variable. The obtained findings are consistent with the literature data [17].

Maximum hip extension phase on both sides was delayed compared with control. The changes were slight albeit statistically significant. We did not find similar data in the available literature.

Decreased swing amplitude in the knee joints is a natural phenomenon; it reflects a decrease in the knee function, which, if compensated, occurs symmetrically. Therefore, we think that these and other changes on the intact side are due to of the fact that compensation requires symmetry. Hence, the observed swing amplitude (A3) asymmetry with the intact limb may be an early sign of developing decompensation and requires further investigation. At the same time, the amplitude delay was symmetrical. Although the GC parameters in patients were significantly higher than in healthy control, the quantitative differences were not important. The delay could be the result of a slower walking pace of the study group. We did not find a description of this phenomenon in the literature.

On the affected side, the first flexion amplitude at the beginning of the stance phase was reduced. The findings are consistent with literature data and may be the result of a more active function of the quadriceps femoris, the principal stabilizer of the joint contributing to its anterior–posterior stability. This mechanism, however, also leads to increased dynamic loads on the joint during this period [26]. We also observed a subsequent decrease in the amplitude of extension in single support phase. Both phenomena are well known and described in the literature [27].

According to the analysis of our own observations and literature data, we can infer that the syndrome of three reduced amplitudes (first and second flexions and extension in single support phase) is the most common knee function response to a pathological condition. This is confirmed by our study, as well as others [24,28,29,30].

The possible mechanism for limiting the knee extension in the single support phase is a low-intensive pain syndrome. As known from clinical practice, knee extension in more manifest clinical cases is associated with pain. It is one of the mechanisms contributing to the development of flexion contracture. Pain makes the patient try to avoid straightening the joint at the beginning and end of the support phase [31]. On the whole, the joint reduces its effective motion range, in both the stance and the swing phases.

Although we did not obtain any statistical proof, there was a decrease in the rotational component in the hip and knee joints on both sides.

According to some authors [16,32], a characteristic feature of the gait in patients with medial gonarthritis is increased adduction in the knee joint in the single support phase. We did not obtain such results in the walking tests in our study. Although we have no statistical proof, there was a decrease in the knee abduction–adduction amplitudes on both sides.

## 6. Conclusions

Thus, the gait of subjects with Grade II medial OA is characterized by several groups of symptoms: the symptoms of the affected limb unloading, the symptoms of limiting the load on the affected joint and the musculoskeletal system as a whole, and the symptoms of gait harmonization.

The symptoms of unloading the affected side include decreased SDS and, partly, increased GC and reduced walking speed (as indirectly inferred from the decreased hip movement amplitude in combination with the increased GC).

The symptoms of limited loading of the affected joint include a reduction in the total motion range of hip and knee on the affected side.

The symptoms of gait harmonization include quite a number of symmetric changes on the healthy side: increased GC, reduced hip and knee amplitudes, and later phases of hip extension and knee swing flexion.

The symptoms of unloading the affected side and those of harmonization are the common symptoms of adaptation, typical for several pathological conditions with a relatively preserved function [33]. Therefore, they are not pathognomonic for Grade II knee OA. The load-limitation symptoms are also common in other pathological conditions of the knee [33]. Hence, we can conclude that the set of functional symptoms associated with Grade II knee OA reflects the problem in a particular knee joint and the compensatory rearrangement of movement patterns.

The intensity of the observed symptoms can help assess changes in the subject’s functional condition over time, during the treatment, as well as to assess treatment outcomes.

Our study findings present some limitations. First, the study did not include patients with other types of gonarthritis of the same grade. The form of gonarthritis with a predominant lesion of the medial compartment is the most common; however, it is not limited to only this.

For the same reason, the research group was relatively small. Potentially, an increase in the group size to include other possible forms of gonarthritis could reveal other functional symptoms of impairment. On the other hand, an increase in the number of studies could lead to a blurring of a symptoms.

We did not have the technical ability to study the movements in the knee joints in the frontal plane, as well as the developed moments of forces during walking. Both parameters are of significant importance precisely in this clinical form of gonarthritis, especially accompanied by overweight, which was typical for the study group. We did not find any functional differences by gender in this study. At the same time, men showed a body mass index higher than women. It is possible that this can be assessed on a larger number of subjects. In this study, we aimed to investigate the typical changes in gait function, as well as hip and knee joints.

## Figures and Tables

**Table 1:** Patient data.

Parameter	Males	Females
Number	13	13
Age (years)	49.7 (39–63)	56 (46–67)
Height (cm)	173 (160–183)	162.5 (153–168)
Weight (kg)	83.2 (62–123)	85.5 (56–104)
Body mass index	27.5 (19.8–36.7)	32.5 (19.8–44.4)
Movement amplitude	0°–120° (1.5°–130°)	0°–116° (0°–125°)
Extension deficit	1.5° (0°–10°)	0°–0.45° (0°–5°)

**Table 2:** Temporal parameters of gait cycle and impact amplitude at the start of the stance phase.

Parameter	Affected	Intact	Control
GC (s)	1.24 [1.1; 1.3] *p* = 0.047	1.245 [1.1; 1.3] *p* = 0.046	1.2 [1.1; 1.2]
SDS (% of GC)	49.7 [49.4; 50.4] *p* = 0.451	49.9 [49; 50.3] *p* = 0.528	50.1 [49.5; 50.3]
Load (g)	1.5 [1.8; 1.4] *p* = 0.101	1.6 [1.8; 1.5] *p* = 0.375	1.7 [1.8; 1.6]

**Table 3:** Hip movement amplitudes and phases.

	Affected	Intact	Control
X1 (% of GC)	4.1 [2.2; 5.3] *p* = 0.674	4.7 [3.7; 7.3] *p* = 0.288	4.3 [1.5; 5.2]
A1 (°)	19.2 [15.5; 23.5] *p* = 0.00005	19.3 [15.9; 23.9] *p* = 0.0004	27.4 [23.1; 29.4]
X2 (% of GC)	58.4 [56.9; 59.7] *p* = 0.001	58.8 [57.1; 60.3] *p* = 0.0001	56.0 [54.2; 57.6]
A2 (°)	−12.5 [−14.6; 8.7] *p* = 0.329	−10.2 [−12.6; −8.8] *p* = 0.842	−10.5 [−13.4; −7.7]
Add. (°)	12.3 [9.5; 17.2] *p* = 0.565	14.3 [9.5; 17.5] *p* = 0.807	13.1 [10.3; 17.6]
Rot. (°)	10.7 [8.4; 13.1] *p* = 0.124	10.2 [6.9; 13] *p* = 0.051	13.0 [8.7; 15.9]

**Table 4:** Knee movement amplitudes and phases.

Parameter	Affected	Intact	Control
X1 (% of GC)	16.7 [15.6; 18.1] *p* = 0.973	17.1 [15.4; 18.7] *p* = 0.912	16.9 [14.9; 18.3]
A1 (°)	17.5 [8.2; 20.2] *p* = 0.039	20.1 [15.3; 22.1] *p* = 0.603	19.1 [17.5; 23.4]
X2 (% of GC)	43.8 [36.9; 46.8] *p* = 0.947	43.7 [41.6; 45.8] *p* = 0.929	44.1 [42.3; 45.2]
A2 (°)	9.6 [6; 12.1] *p* = 0.05	9.8 [7.5; 12.6] *p* = 0.019	5.4 [3.7; 10.8]
X3 (% of GC)	75.8 [74.4; 76.6] *p* = 0.002	76.5 [75.1; 77.5] *p* = 0.00002	74.2 [73.1; 75.1]
A3 (°)	67.9 [64.5; 72.2] *p* = 0.025	65.9 [59.8; 69.9] *p* = 0.0709	68.3 [65.7; 72.2]
Add. (°)	11.8 [8.7; 18.6] *p* = 0.037	10.95 [8.4; 15] *p* = 0.005	15.6 [12.0; 23.9]
Rot. (°)	16.25 [12.9; 20.5] *p* = 0.072	16.3 [13.2; 22] *p* = 0.195	19.9 [14.9; 23.9]

## Data Availability

All primary data are with the authors of this study.
